# Combined MR Volumetry and T2* Relaxometry Reveals the Olfactory System as an Iron-Dependent Structure Affected by Radiation

**DOI:** 10.3390/neurolint17040053

**Published:** 2025-04-08

**Authors:** Njenga R. Kamau, Michelle R. Tamplin, Chu-Yu Lee, Eric D. Axelson, Isabella M. Grumbach, Michael S. Petronek

**Affiliations:** 1 Department of Radiation Oncology, University of Iowa, Iowa City, IA 52242, USA; 2 Department of Internal Medicine, University of Iowa, Iowa City, IA 52242, USA; 3 Department of Radiology, University of Iowa, Iowa City, IA 52242, USA; 4 Department of Psychiatry, University of Iowa, Iowa City, IA 52242, USA

**Keywords:** radiation-induced brain injury, iron metabolism, T2* mapping, olfactory system

## Abstract

**Background/Objectives:** Radiation therapy can often lead to structural and functional changes in the brain resulting in radiation-induced brain injury. This study investigates the MRI-detectable effects of whole-brain irradiation across all neuroanatomical structures in adult mice, with a specific focus on T2* MRI measurements, to evaluate regions that may be particularly sensitive to iron accumulation. **Methods:** One year following irradiation or sham treatment, mice were imaged with a 7T MRI to evaluate changes in regional volume and T2* relaxation times across more than 652 neuroanatomical using the DSURQE mouse brain atlas. **Results:** Statistical analysis identified 301 altered regions with respect to regional volume and 85 regions with respect to T2* relaxation showing significant differences relative to the control group (*p* < 0.05). Further data refinement, including the consolidation of redundant, bi-lateral structures revealed 18 subregions with significant changes in both volume and T2*. The data refinement revealed that the most represented system was the olfactory system (8/18 regions, 44%). The olfactory regions also showed the most pronounced changes and greatest correlation between the two metrics. **Conclusions:** These findings are suggestive that ionizing radiation may cause a pronounced disruption in the olfactory system that coincides with potential iron accumulation.

## 1. Introduction

Radiation-induced brain injury (RIBI) is a common late adverse effect of radiation exposure to the head and neck, characterized by neuroanatomical changes and cognitive impairment [1]. Whole brain radiation therapy (WBRT) is considered a standard therapy for cases of multiple brain metastases; however, up to 50% of patients will develop significant cognitive impairment 6–12 months after the completion of therapy [1,2]. It is well established that radiation can lead to the degeneration of critical structures (e.g., hippocampus) resulting in long-term cognitive impairment. This has led to the introduction of hippocampal avoidance in patients receiving WBRT [3]. Beyond this technical approach, there are still limited therapeutic options to mitigate the cognitive impairments associated with RIBI. The current best option for mitigating RIBI is memantine, an FDA-approved drug that is used as a neuroprotective agent in Alzheimer’s disease [4], but there is still a critical need for more efficacious therapeutic options. To bring a novel therapeutic approach, a more complete understanding of the mechanisms driving RIBI is needed.

The robust generation of oxidants (i.e., reactive oxygen species, ROS) is a main feature of ionizing radiation and, thus, damage associated with oxidative stress (e.g., ferroptosis) is thought to be central to RIBI [5]. Following the generation of ROS, iron can serve as a catalyst of damage [6], especially through its accumulation in damaged neurons [7]. Iron accumulation is thought to be a major underlying pathologic feature of Parkinson’s and Alzheimer’s disease, among several other neurodegenerative disorders [7]. However, the role of iron in radiation-induced neurodegeneration remains unclear and necessitates more robust investigation. Therefore, utilization of advanced imaging to assess the chronic effects of ionizing radiation on regional brain iron metabolism may provide significant insights into RIBI.

Magnetic Resonance Imaging (MRI) can play a useful role in evaluating neurodegenerative processes, particularly through advanced techniques like T2* imaging. T2*-based MRI is a quantitative approach that assesses local magnetic field variations caused by changes in the paramagnetic properties of the region of interest (e.g., iron deposition) [8]. Conventionally, T2* mapping has been used to evaluate changes in tissue iron content, particularly with respect to liver and heart damage associated with iron overload [9]. T2* mapping has shown promise in detecting iron accumulation in neurodegenerative cases such as Parkinson’s disease where changes in T2* relaxation have been shown to correlate with disease progression, but is still under investigation for this application [10]. Therefore, it can be hypothesized that T2* mapping is a useful tool to assess iron deposits following exposure to ionizing radiation. This type of analysis can provide novel insights into potential iron-dependent brain regions that play an important role in the onset of RIBI. The primary focus of this study was to explore changes in T2* relaxation time across the entire murine brain to interrogate neuroanatomical regions that may be particularly sensitive to iron accumulation following exposure to ionizing radiation.

## 2. Materials and Methods

### 2.1. Animal Care and Irradiation Procedure

**Figure 1:**
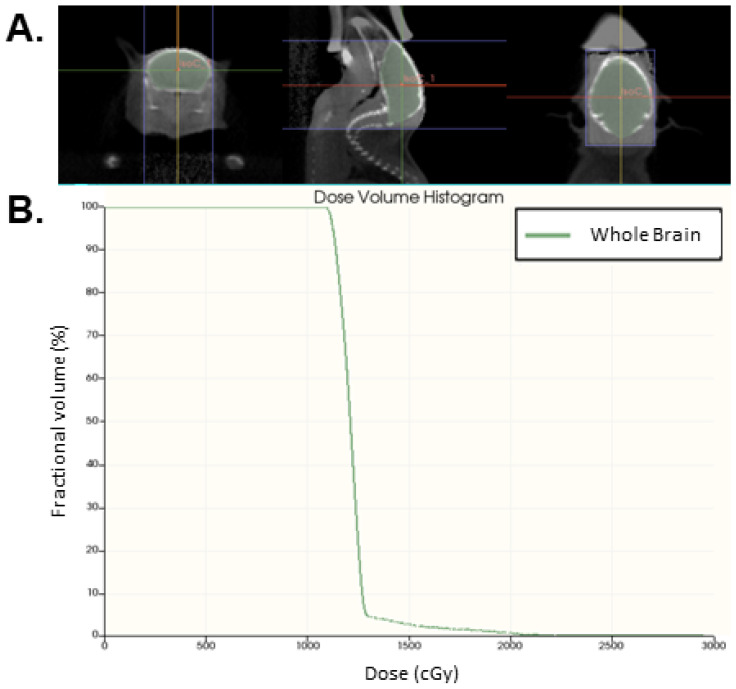
Whole brain radiation treatment plan. (**A**) Representative axial (right panel), sagittal (middle panel), and coronal (left panel) cone-beam CT images with the whole brain contoured in green for dose confirmation. An amount of 12 Gy was prescribed to the isocenter (IsoC_1) using a 12 × 10 mm field size (within the purple lines). The dose delivered to the whole brain was confirmed by dose volume histogram (**B**).

All experiments were approved by the Institutional Animal Care and Use Committees (IACUC) at the University of Iowa (IACUC Protocol #2112263) and performed in compliance with the Institute of Laboratory Animal Resource, National Academy of Science. At 10 weeks of age, male C57Bl/6J mice underwent sham treatment (*n* = 5) or whole brain irradiation (IR; *n* = 5) using the Xstrahl Small Animal Radiation Research Platform (SARRP). Mice were sedated with isoflurane prior to and during the procedure. Prior to irradiation, mice underwent a cone beam CT for treatment planning purposes. A mean dose of 12 Gy was prescribed to a single isocenter in the center of the brain (Figure 1A) using a 12 × 10 mm field size. The delivery of this dose was confirmed by contouring the brain volume in MuriPlan (v.3.0.0) and calculating the dose volume histogram (Figure 1B).

### 2.2. Magnetic Resonance Imaging (MRI) Data Acquisition and Processing

**Figure 2:**
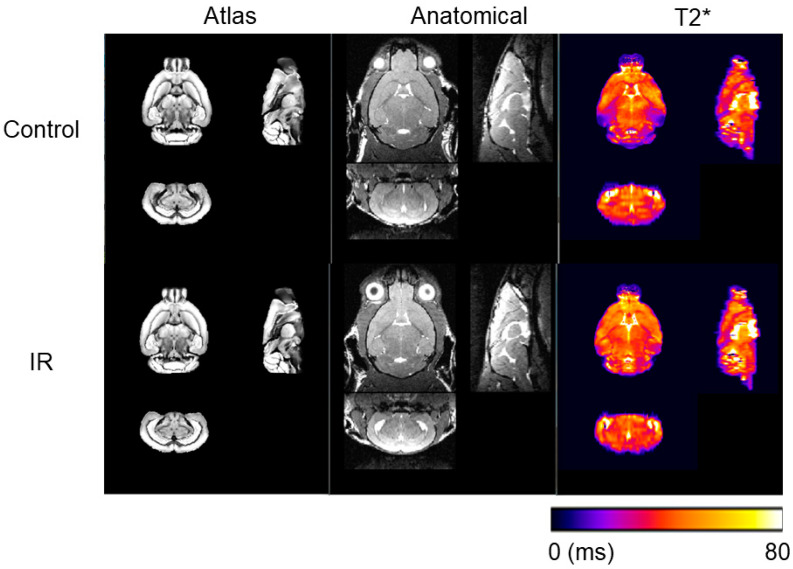
Representative MR images. 12 months following irradiation (IR) or sham (“Control”) treatment, mice were scanned using a GE 7T small animal MRI to generate T2-weighted anatomical images using a 3D-FIESTA sequence and a 2D multi-echo gradient echo sequence was used to produce T2* maps. The DSURQE mouse-brain atlas was used to provide a regional atlas for quantitative analysis.

12 months after sham treatment or IR, mice were imaged on a 7T Discovery MR901 system (GE Healthcare, Milwaukee, WI, USA) using a body transmit coil and a two-channel mouse brain receiver coil to generate anatomical T2-weighted MRI and T2* maps (Figure 2). Prior to image acquisition, a high-order B0 shimming routine was applied to smooth B_0_ field inhomogeneities. A 3D FIESTA sequence was applied for anatomical T2-weighted images (in-plane resolution of 104 µm^2^, slice thickness = 160 µm, pixel bandwidth = 326 Hz, flip angle = 30°, TE/TR = 3/6.1 ms, number of averages = 4, and scan time of 9 min and 28 s). A 2D multi-echo GRE sequence was used for T2* measurements (in-plane resolution of 156 µm^2^, slice thickness = 500 µm, 18 axial slices, pixel bandwidth = 244 Hz, flip angle = 60°, TR = 1000 ms, 6 TEs of 2.5–22.5 ms in increments of 4 ms, number of averages = 2, and scan time of 4 min and 24 s). A two-stage fitting procedure was applied to the GRE magnitude images to generate T2* maps as previously described in Lee, C.-Y. et al. [11] After T2* map generation, brain regions were defined using the structural labels of the DSURQE Mouse Brain atlas images [12]. T2-weighted images for each session were reoriented to a standard orientation using AFNI [13]. Brain extraction was performed with Rapid Automatic Tissue Segmentation [14]. Manual correction by an expert rater of the brain mask was needed in most cases to correct and improve the automated output. T2-weighted images were then normalized to a standard space and all registrations consisted of rigid, affine, and nonlinear (symmetric normalization) components that were conducted using Advanced Normalization Tools [15]. Registration transforms were used to apply the DSURQE whole brain atlas labels to native space for volumetric analysis. The T2* maps were then linearly co-registered to the individual anatomical T2-weighted images and associated DSURQE Mouse Brain atlas structures. All image post-processing was performed by a blinded observer.

### 2.3. Statistical Analysis

**Figure 3:**
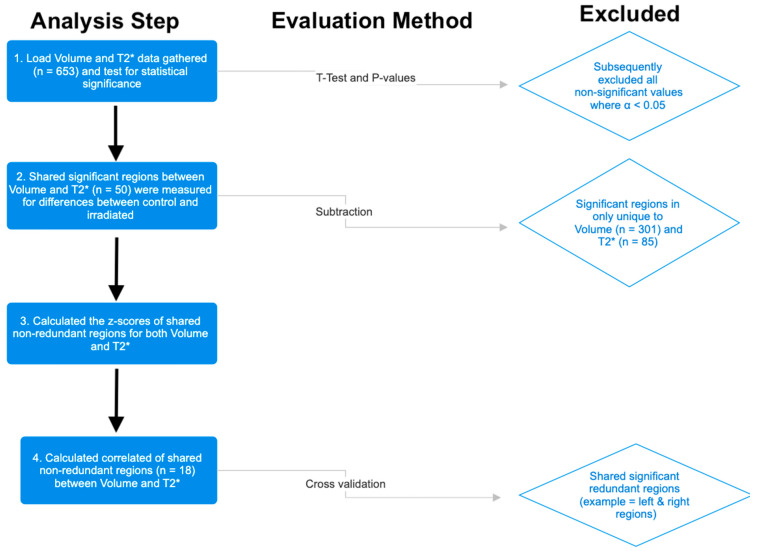
Data analysis pipeline used to identify non-redundant, shared neuroanatomical regions that showed significant differences with respect to changes in volume and T2*.

A custom Python 3.10.9 script within Visual Studio Code (Version 1.85.0) was used to analyze volume and T2* relaxation time on a regional level. For each structure contained within the DSURQE atlas (*n* = 652), significant differences in volume (*n* = 301) and T2* relaxation time (*n* = 85) between control and IR groups were identified using an unpaired *t*-test (implemented using SciPy.stats.ttest_ind). A *p* value less than 0.05 was considered significant. Coincident structures that were significant with respect to volume and T2* were kept for further analysis (*n* = 50). Because the whole brain was irradiated, sided structures (e.g., right hippocampus, left amygdala) were consolidated to further refine the total number of unique independent structures (*n* = 18). Total gray matter, total white matter, and whole brain changes were excluded from this analysis as this study is primarily focused on regional changes as opposed to gross anatomical changes. A summary of the data refinement strategy is shown in Figure 3. The arithmetic difference between regional measures in control and IR-treated mice was determined by subtracting the mean control value from the mean IR value. For each region, the difference in means was divided by the standard deviation of the control population to yield a z-score, with a negative z-score indicating a decrease in volume or T2* relaxation time relative to the control group. To determine the relationship between T2* relaxation time and volume, Pearson correlation coefficients between the z-scores for each metric were calculated for regions that showed significant differences by both measures (*n* = 18).

## 3. Results

**Figure 4:**
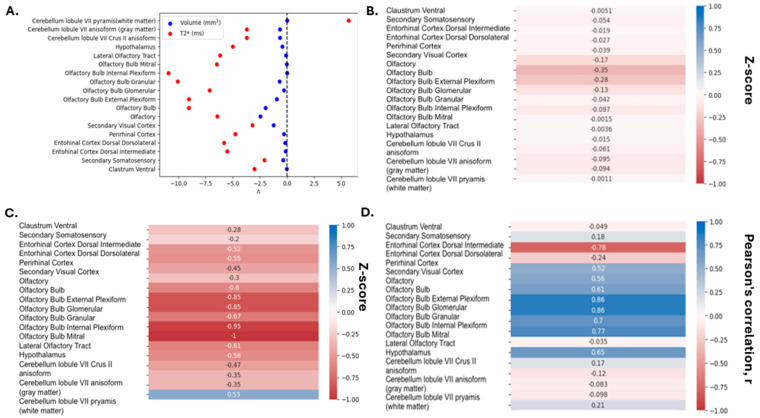
Analysis of neuroanatomical changes in volume and T2*. (**A**) Change in volume and T2* relaxation time in shared neuroanatomical regions that were statistically significant 12 months following IR or sham treatment (*n* = 18). (**B**,**C**) Heat map representation of Z-scores for volume (**B**) and T2* relaxation time (**C**) in shared neuroanatomical regions that were statistically significant 12 months following radiation (*n* = 18). (**D**) Heat map representation of correlation coefficients between change in volume and T2* relaxation time.

Of the original 652 regions outlined by the DSURQE atlas, there was a significant decrease in the volume of 301 regions and a significant change in T2* relaxation time of 85 regions. Following data refinement to identify those regions that were significantly different with respect to both volume and T2* (including the removal of bilateral redundancies), 18 regions showed significant differences by both measures (Figure 4A). These regions include structures in the cerebrum, cerebellum, hypothalamus, cortex, and olfactory regions. Following z-score calculations, there were negative changes with respect to both volume (Figure 4B) and T2* relaxation time (Figure 4C), indicating a potential neurodegenerative effect associated with iron accumulation. By both measures, the most common and largest changes (most negative z-scores) were observed in structures associated with the olfactory system (8/18, 44%). Furthermore, the strongest correlations between volume and T2* relaxation time were also observed in olfactory system regions (Figure 4D). Therefore, it appears as if the olfactory system may be particularly sensitive to volumetric loss by ionizing radiation that is coincident with T2*-detectable changes in iron content.

## 4. Discussion

The goal of this study was to explore long-term changes in T2* relaxation time alongside volumetric changes to identify structures that may be particularly sensitive to iron accumulation following exposure to ionizing radiation. After evaluating 652 total neuroanatomical structures, we were able to isolate 18 substructures that showed significant volume loss and decrease in T2* relaxation. In the irradiated mice, a T2* signal loss was observed, indicating potential iron accumulation, as it is well established that increased iron content causes a decrease in T2* relaxation [9]. The relevant neuroanatomical structures observed include the cerebellum, entorhinal cortex, visual cortex, somatosensory cortex, claustrum, and olfactory system. The most notable and robust changes in volume and T2* relaxation occurred within the olfactory system (8/18, 44%). These findings suggest that the olfactory system may be particularly sensitive to radiation-induced iron metabolic disruptions. It is important to note, however, that the small volume of the olfactory bulb and its encapsulation within the surrounding bone lends itself vulnerable to potential magnetic susceptibility artifacts and should be interpreted carefully. Beyond T2* mapping, the additional use of quantitative susceptibility mapping may provide more robust iron content analysis as this approach may be more sensitive to changes in iron content and has previously been employed to evaluate iron accumulation in neurodegenerative processes [16,17,18,19]. Therefore, we would recommend that further biological validation of the hypothesis by directly measuring iron accumulation within the olfactory bulb as compared to the rest of the brain should be completed.

The olfactory bulb, located above the nasal cavity, is particularly vulnerable to radiation exposure during treatments for head and neck cancers [20]. For example, 12 patients with nasopharyngeal carcinoma treated with radiotherapy all showed a robust temporal increase in olfactory threshold during therapy that persisted for at least a month following the completion of radiation [21]. These patients commonly reported imbalances in their olfactory perception, with a heightened sensitivity to unpleasant odors and a distortion of taste, such as foods tasting more bitter. One study assessed 44 patients undergoing chemotherapy for head and neck cancer over a six-week period, with evaluations conducted every two weeks. Olfactory identification was measured using the Scandinavian Odor Identification Test (SOIT), which involves 16 different odorants [22]. The results showed that radiation to the olfactory epithelium led to a decline in both detection sensitivity and odor identification. Pre-clinically, it has also been shown that mouse brain irradiation can acutely disrupt olfactory sensation that worsens over time [23]. Early disruptions in olfactory function have also been observed in patients with Parkinson’s disease that precede the most notable neuromotor disruptions that can be attributed to iron accumulation in the basal ganglia [24]. Of important note, the olfactory bulb is a primary site of adult neurogenesis as neuronal precursor cells will migrate from the subventricular zone through the olfactory bulb [25,26]. Thus, initial damage to the olfactory bulb may be a critical step that preludes the long-term cognitive decline associated with radiation exposure. In support of this hypothesis, focal radiation of the olfactory bulb in pediatric mice resulted in off-target volumetric loss in the anterior commissure and subventricular zone, indicating that radiation-induced olfactory damage may result in impaired neurogenesis and volume loss at distal brain regions [27]. Taken together, it can be hypothesized that early iron accumulation in the olfactory system may precede the cognitive decline associated with RIBI, which warrants more robust mechanistic interrogations (e.g., direct interrogation of iron content in the olfactory bulb).

## 5. Conclusions

This study has leveraged T2* mapping as an iron-dependent imaging metric to interrogate potential neuroanatomical structures that are particularly sensitive to iron-mediated damage associated with radiation-induced neurodegeneration. By assessing coincidence changes in regional volume and T2* relaxation 12 months following irradiation, we have been able to identify 18 specific structures where 8 of these neuroanatomical structures (44%) affected by radiation were contained within the olfactory system. Taken together, these data suggest that the olfactory system may be a particularly iron-sensitive structure that can influence the progression of RIBI. Future studies should be directed to explore the role of olfactory iron accumulation as an underlying contributor to RIBI.

## Data Availability

The data presented in this study are available upon the request of the corresponding author.
